# Enrichment Characteristics of Cr in Chromium Slag after Pre-Reduction and Melting/Magnetic Separation Treatment

**DOI:** 10.3390/ma14174937

**Published:** 2021-08-30

**Authors:** Shaoyan Hu, Deyong Wang, Xianglong Li, Wei Zhao, Tianpeng Qu, Yun Wang

**Affiliations:** 1 School of Iron and Steel, Soochow University, Suzhou 215137, China; syhu616@suda.edu.cn (S.H.); deyongwang1222@163.com (D.W.); tianpengqu8119@163.com (T.Q.); 2 China ENFI Engineering Co., Ltd., Beijing 100038, China; yunwang8901@126.com

**Keywords:** chromium slag, chromium–iron ratio, pre-reduction, melting separation, magnetic separation

## Abstract

Concentrating the chromium in chromium slag and improving the chromium–iron ratio is beneficial for the further utilization of chromium slag. In this paper, chromium slag obtained from a chromite lime-free roasting plant was used as the raw material. Pellets made of the chromium slag and pulverized coal were reduced at different pre-reduction temperatures and then separated by a melting separation process or magnetic separation process, respectively. The mass and composition of the metallized pellets before separation, along with the alloy and tail slag after separation, were comprehensively analyzed. The experimental results showed that the output yield of alloy, iron recovery rate, and chromium content in the alloy were all higher when using melting separation than when using magnetic separation, because of the further reduction during the melting stage. More importantly, a relatively low pre-reduction temperature and selection of magnetic separation process were found to be more beneficial for chromium enrichment in slag; the highest chromium–iron ratio in tail slag can reach 2.88.

## 1. Introduction

Chromium has been widely applied in many industrial processes, such as leather tanning, electroplating, and mineral extraction. It is also an important ingredient in protective coatings, especially for stainless steel.

Chromium slag is the waste residue generated by the industrial process of extracting chromium (Cr) from chromite ore. Due to different needs for chromite quality in the target product, the extraction processes for chromium (Cr) are also different, leading to diverse compositions and properties for chromium slag [1,2,3]. Chromium salt manufacturing is a typical extraction process, which takes chromite, sodium carbonate, and dolomite as raw materials. After high-temperature oxidation roasting, chromium oxides are transformed into water-soluble sodium chromate, and the remaining tailings become chromium slag after water leaching [4,5]. However, about 10% of the chromium remains in the chromium slag and contains water-soluble, migratory, and carcinogenic Cr^6+^, which is harmful to the environment [6,7,8]. If it is not recycled or reused, not only are resources wasted, but serious pollution of the environment results, which will have a serious impact on the health of the surrounding residents because of the toxic Cr^6+^ content of the slag [9,10,11]. At present, the treatment method for chromium slag is mostly landfill after reduction or solidification, but there are still potential environmental hazards and resource waste to consider [12]. Thus, it is valuable to explore further treatment methods for chromium slag.

According to the flux used in the roasting process, the chromium salt manufacturing process can be divided into the lime-based roasting process and the lime-free roasting process [13]. The chromium slag used in this research was obtained from a chromite lime-free roasting plant. The main components of the lime-free roasting slag are iron oxide and chromite oxide. Based on the chemical composition characteristics of the lime-free roasting slag, the recovery and utilization methods for metallic elements mainly include the hydrometallurgical method and pyrometallurgical method. The hydrometallurgical extraction method generally uses highly corrosive acids as extractants, such as sulfuric acid and hydrochloric acid, to achieve the transfer of metallic elements from the chromium slag to the liquid phase [14]. However, the selectivity of the acid-leaching process is poor, because there is no difference in the recovery of various metallic elements from the slag. A lot of subsequent separation steps need to be added to achieve real recycling [15]. In addition, efficient and low-cost treatment of the waste acids is also a problem.

The advantage of pyrometallurgical extraction lies in its excellent selectivity. By adding the reducing agent and slagging agent and adjusting the processing temperature and operating pressure, it can achieve the selective recovery of different metallic elements. The principal elements in chromium slag are iron and chromium. Reduction by the proper amount of carbon can convert toxic Cr^6+^ into nontoxic chromium oxides, such as CrO and Cr_2_O_3_; meanwhile, the iron oxide can be reduced partly to metallic iron [16,17,18]. After the reduction treatment, the metallic iron can be separated from the chromium slag by the melting separation method or magnetic separation method because of the magnetic difference between the metal and slag [19,20,21,22]. According to extensive experimental research and productive practices, the higher the ratio of chromium to iron (Cr/Fe) in slag, the higher the economic value of the slag. In reality, only when the mass ratio of chromium to iron (Cr/Fe) in the slag is greater than 2.0 can the slag meet the requirements for further use, which is mainly the production of ferrochromium. [23]

In order to improve the mass ratio of chromium to iron (Cr/Fe) in slag, it is necessary to extract iron from chromium slag and retain chromium in chromium slag. Compared with other synthesis techniques, carbothermal reduction is the most widely used method because of its low cost and simple process. From thermodynamic data on the reduction reactions of iron oxides and chromium oxides, as shown in Table 1 [24], it is clear that iron oxides can be more easily reduced by carbon than chromium oxide. The starting temperature of chromium oxide (Cr_2_O_3_) reduction is 1521.0 K, which is much higher than the reduction temperature of iron oxide, which is only 994.9 K. Thus, it is practicable to control the extraction of iron and the retention of chromium by adjusting the reduction temperature.

However, after the controllable reduction, the reaction products need to be separated into the metallic alloy and non-metallic slag. Melting separation and magnetic separation are the most widely applied and mature separation processes. Selection of the separation process also has a significant impact on the yield and composition of the final product.

In this paper, chromium slag obtained by chromite lime-free roasting was used as the raw material, and the experimental processes of pre-reduction, followed by melting/magnetic separation, were adopted to deal with the chromium slag. The main purpose of this paper was to investigate the chromium enrichment characteristics of chromium slag under different pre-reduction temperatures and different separation processes, with the aim of achieving a higher mass ratio of chromium to iron (Cr/Fe) in the final tail slag.

## 2. Experiment

### 2.1. Raw Materials

As mentioned above, the chromium slag was obtained from a chromite lime-free roasting plant. As the key raw material of the experiment, it was necessary to obtain the specific composition of the chromium slag. The slag was ground to a particle size of less than 0.074 mm first; then, the powder slag was sent to National Analysis Center for Iron and Steel China for accurate composition analysis. T.Fe, T.Cr, Al_2_O_3_, MgO, CaO, and SiO_2_ were clearly identified for analysis. The T.Fe and T.Cr contents were detected by oxidation–reduction titration method, and the Al_2_O_3_, MgO, CaO, and SiO_2_ content were detected by wavelength dispersive X-ray fluorescence spectrometry method. The composition of the dry chromium slag used in the experiment is shown in Table 2. The T.Fe content and T.Cr content were relatively high, indicating resource utilization potential. Considering that the chromite was roasted in an oxidizing atmosphere, it was assumed that the iron in the slag existed in the form of Fe_2_O_3_, and the chromium in the slag existed in the form of Cr_2_O_3_. The Al_2_O_3_ content, MgO content, and SiO_2_ content in the chromium slag were similar, while the CaO content was quite low, which is a typical feature of the chromite lime-free roasting process. In addition, it should be noted that the total content of the above components did not reach 100%. Apart from the above components, chromium slag also contains a small amount of natrium salt, titanium oxide, phosphorus oxide, and other gangue minerals. It may even include crystal water that has not been completely removed during drying. Due to their small effect on the chromium enrichment characteristics, quantitative analysis of these components was not performed.

In addition to the chromium slag, another main raw material of the experiment was pulverized coal, which played the role of reducing agent. Before analyzing the composition, the pulverized coal was ground to a particle size of less than 0.074 mm, the same as the chromium slag. Proximate analysis of the pulverized coal showed that the fixed carbon content was 85.66 wt.%, the volatile content was 1.59 wt.%, and the ash content was 12.13 wt.%.

In this research, an analytically pure SiO_2_ reagent was used to adjust the basicity of the pellets. The SiO_2_ content in the analytical reagent was more than 99.5 wt.%.

### 2.2. Experimental Schemes

In order to reveal the reduction and separation behaviors of chromium and iron in slag, four experiments with different pre-reduction temperatures and different separation processes were designed and carried out in this research, as shown in Table 3. Choosing 1373 K and 1523 K as the pre-reduction temperatures were mainly based on the experiment results of Cheng G et al. [25], who found that 1373 K was the optimal reduction temperature for reducing iron from slag under their experimental conditions.

Experiment NO. 1 was pre-reduced at 1373 K, followed by a melting separation step, whose separation temperature was 1853 K, with a separation processing time of 60 min. Experiment NO. 2 was pre-reduced at 1523 K, followed by a melting separation step, whose separation temperature was also 1853K, and the separation processing time was 60 min. Experiment NO. 3 was pre-reduced at 1373 K, followed by a magnetic separation step. Experiment NO. 4 was pre-reduced at 1523 K, followed by a magnetic separation step. Uniformly, the pre-reduction time was 45 min for all experiments. In order to reduce the experimental error, each group experiment was repeated three times.

**Figure 1:**
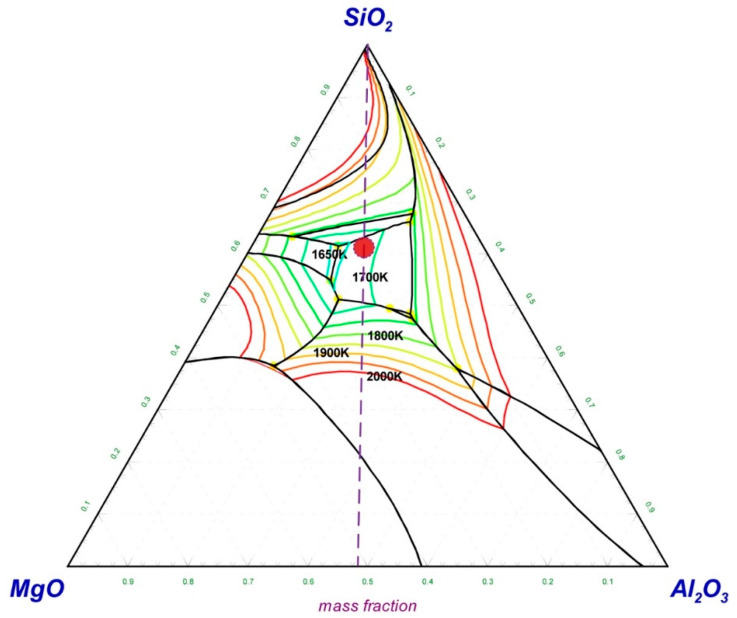
MgO–SiO_2_–Al_2_O_3_ phase diagram with isotherm lines for *p* = 1 atm, drawn with FactSage 7.2.

Generally, the melting point of the slag has a major influence on the separation of the alloy and slag during the melting separation process. Low-melting-point slag is beneficial for the separation of the metallic alloy from the tail slag. In order to figure out the appropriate slag composition, the MgO–SiO_2_–Al_2_O_3_ phase diagram was calculated and is shown in Figure 1. The phase diagram was calculated and drawn by the thermodynamic software FactSage 7.2, which is developed by Thermfact/CRCT (Montreal, Canada) and GTT-Technologies (Aachen, Gemany). During the phase diagram calculation, the selected database was FToxide, and the operating pressure was 101,325 Pa. The isotherm lines from 1650 K to 2000 K were plotted in the phase diagram with an interval of 50 K. The calculated phase diagram was compared with the slag atlas [26] and was validated by it. According to the phase diagram of MgO–SiO_2_–Al_2_O_3_ in Figure 1, the slag composition in the low-melting-point area was around *m_SiO_*_2_:*m_Al_*_2*O*3_:*m_MgO_* ≈ 6:2:2, while the initial slag composition shown in Table 2 was *m_SiO_*_2_:*m_Al_*_2*O*3_:*m_MgO_* ≈ 1:1:1. In order to obtain the target slag composition (*m_SiO_*_2_:(*m_Al_*_2*O*3_ + *m_MgO_*) = 6:4) with a low melting point, 18.38 g SiO_2_ should be added to every 100 g of chromium slag. As a result, when the melting separation method was adopted, the basicity of the slag ((*m_CaO_* + *m_MgO_*)/*m_SiO_*_2_) was reduced to 0.36 from the initial value of 0.99 due to the addition of SiO_2_. Because the melting properties of the slag have little effect on the magnetic separation process, there is no need to add SiO_2_ into the pellets when using the magnetic separation method, whose basicity remains at the initial value of 0.99.

As pulverized coal is the reducing agent of chromium slag, its addition amount in the pellets is very important for reduction reactions. Since the chromium slag came from a chromate roasting process, which was in an oxidizing atmosphere, it was assumed that the iron and chromium in the chromium slag would exist in the form of Fe_2_O_3_ and Cr_2_O_3_ when they took part in the carbothermal reduction reaction and that the gaseous product of the reduction reaction would be CO. The concentration of the added carbon powder was expressed as the mole ratio of carbon to reducible oxygen (*n_C_*/*n_O_*), instead of mass %, in order to meaningfully represent the significance of the carbon addition. In the (*n_C_*/*n_O_*) value, reducible oxygen (*O*) is the total amount of oxygen present in the form of Fe_2_O_3_, while excluding the oxygen in Cr_2_O_3_. The reason for excluding the oxygen in Cr_2_O_3_ was that the purpose of this research was to reduce the iron and retain the chromium as much as possible. In order to ensure that the iron oxide was fully reduced, the addition of coal needed to be in excess, and the carbon–oxygen ratio of the experiment was *n_C_*/*n_O_* = 1.1.

### 2.3. Experimental Procedures and Devices

**Figure 2:**
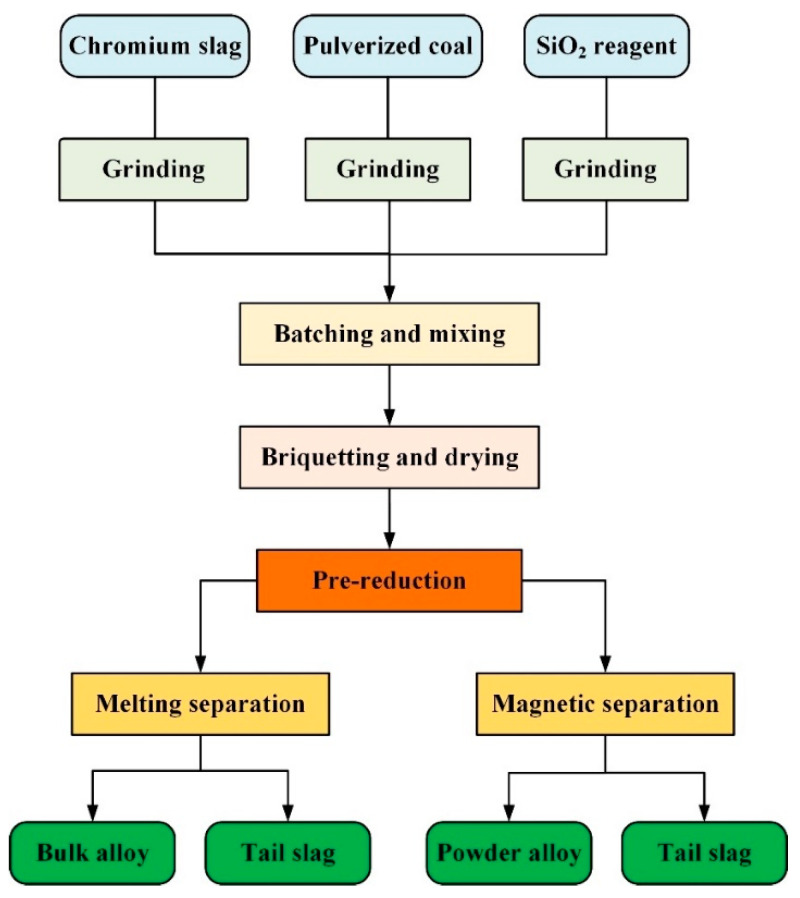
Experimental procedures for the pre-reduction and separation processes.

**Figure 3:**
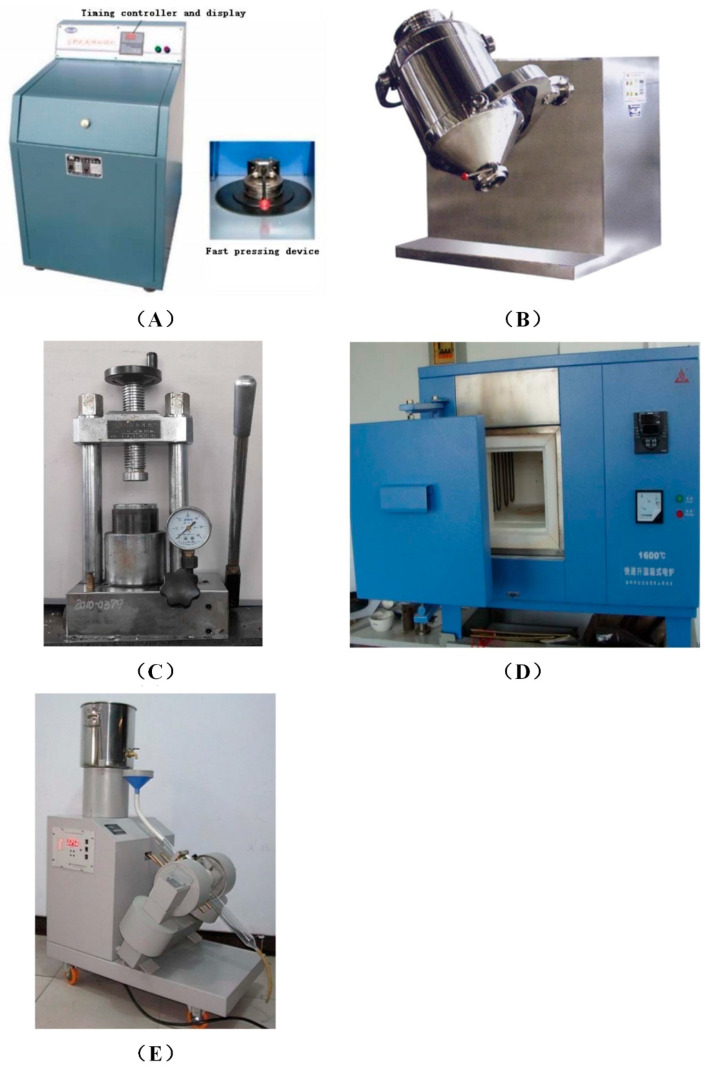
Experimental devices. (**A**) Grinder; (**B**) mixer; (**C**) presser; (**D**) muffle furnace; (**E**) magnetic separator.

The experimental procedures are shown in Figure 2, including grinding, batching, briquetting, pre-reduction, and separation. Details of every procedure are described in combination with the relevant devices. The main devices used in the experiment were a grinder, mixer, presser, muffle furnace, and magnetic separator, as shown in Figure 3.

#### 2.3.1. Grinding

As mentioned above, both the chromium slag and the pulverized coal were ground to a particle size of less than 0.074 mm first, not only for the composition analysis, but also for the subsequent batching. Crushing and grinding of the raw materials were performed in a grinder, as shown in Figure 3A. The grinder was suitable for the preparation of powdered samples of coal, ore, slag, and other raw materials. After the motor started, the eccentric block was driven to rotate at high speed to drive the exciting platform to generate the exciting vibration. After loading the material into the material bowl, the material collided with the crushing rod and the crushing ring in the material bowl strongly, then was crushed into very fine grains. After grinding, the ground powder was sieved with a 200-mesh sieve. If the particle size did not meet the requirements, the grinding time was prolonged.

#### 2.3.2. Batching and Mixing

The ground chromium slag, pulverized coal, and silica were weighed and mixed in specific proportions corresponding to the respective experimental schemes. In order to achieve uniform mixing, each material was put into a 3-dimensional motion mixer, as shown in Figure 3B. During the mixing operation, various materials were well mixed due to the multi-directional rotation of the mixing tank, while avoiding the stratification phenomenon caused by gravity in general mixers.

#### 2.3.3. Briquetting and Drying

Each group’s well-mixed raw material was put into the presser for briquetting. The presser (Figure 3C) compacts powder samples. It can press granular material into tablets or granules. It is suitable for pressing tablets, catalysts, and metal powders in the laboratory. By manually pressing the lever, the oil can be pressed from the oil pool into the cylinder below the mold. The one-way valve prevents the oil from returning to the oil pool, so the high pressure of the oil cylinder can be maintained, and the maximum pressure is 60 MPa. The powder materials in the mold were molded by high pressure, and the molded pellets were obtained after demolding. The obtained pellets had an approximate diameter of 20 mm and mass of 10 g.

Before the pre-reduction step, the pellets were dried at 378 K for 4 h in an oven.

#### 2.3.4. Pre-Reduction

Pre-reduction of the pellets was carried out in a muffle furnace, as shown in Figure 3D. This is a high-temperature resistance furnace with a maximum temperature of 1873 K. The furnace temperature was measured by a double platinum-rhodium thermocouple and controlled by the program. According to the experimental scheme, pre-reduction temperatures of 1373 K (Experiment NO. 1 and NO. 3) and 1523 K (Experiment NO. 2 and NO. 4) were used. When the muffle furnace reached the required temperature (1373 K or 1523 K), the graphite crucible containing the pellets was put into the furnace. Then, 45 min after the reduction, the graphite crucible was taken out and the pellets were covered by another graphite crucible to prevent oxidation. The cooled metallized pellets were used in subsequent experiments for melting separation or magnetic separation.

#### 2.3.5. Melting Separation

The temperature of the muffle furnace was raised to 1853 K, and then the crucible containing the metallized pellets was put into the furnace. The temperature was maintained at 1853 K for 60 min, and then the crucible containing the sample was removed from the furnace and covered by a graphite plate to prevent oxidation during cooling. After the sample was cooled, the bulk alloy was separated from the slag, and then the slag and alloy were sent for chemical analysis.

#### 2.3.6. Magnetic Separation

Magnetic separation was carried out with a magnetic separator, as shown in Figure 3E. The magnetic separator can generate a strong magnetic field at the middle position of the magnetic separator tube, and the maximum magnetic field intensity is 500 mT. Metallized pellets obtained by pre-reduction were ground to a particle size of less than 0.148 mm. Then, the powder was mixed into water and stirred, before being passed through the magnetic separation tube with a magnetic field intensity of 200 mT, so that the magnetic powder remained in the magnetic field in the magnetic separator tube, and the non-magnetic slag passed through the tube with the water. After all of the mixture of water and powder had been poured into the magnetic separation tube, water continued to be poured into the tube until the flowing water became clear. Then, the magnetism of the magnetic separator was switched off, and the alloy in the magnetic separation tube was flushed with water into another container until the magnetic separation tube was clean. The sample of tail slag was filtered, and the residue on the filter paper was collected and dried for 4 h to obtain dry tail slag; dry alloy powder was obtained in the same way. Finally, the dry tail slag and dry alloy powder were sent for chemical analysis.

## 3. Results and Discussion

After finishing the experiments, the metal distribution between the slag and the alloy, the metal recovery rate, and the mass ratio of chromium to iron (Cr/Fe) in final tail slag were compared in detail. Based on the experimental results, the proper process route and technological parameters for the recovery of chromium slag were explored.

Table 4 shows the weight and composition of the metallized pellets before separation, together with the alloy and tail slag after separation. Through pre-reduction and melting/magnetic separation, iron and chromium are distributed both in the alloy and in the tail slag. The multiple indicators, such as the output yield of alloy, the distribution ratio of metals (iron and chromium) in the slag and alloy, the recovery rate of metals (iron and chromium), and the mass ratio of chromium to iron (Cr/Fe) in the slag and alloy, can quantitatively express the effect of the process route and technological parameters on the chromium enrichment characteristics.

### 3.1. Output Yield of Alloy

The output yield of alloy is defined as *φ*.

*φ* = *m_a_/m*_0_ × 100% (1)

where *m_a_* is the mass of the obtained alloy, in g, and *m*_0_ is the mass of the metallized pellets before separation, in g.

**Figure 4:**
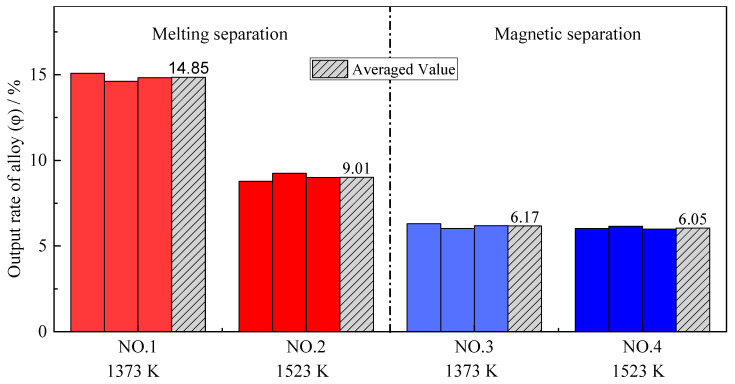
Output yield of alloy with different pre-reduction temperatures and different separation processes.

Previous research showed that the addition of a reducing agent had a dominant effect on the output of alloy with sufficient time and at an appropriate temperature [27,28]. As shown in Figure 4, the magnetic separation processes at different reduction temperatures have almost the same alloy output yield. However, the output of alloy from the melting separation process is significantly higher than that of the magnetic separation process. Because the melting process was carried out in the graphite crucible, the residual iron oxides and chromium oxides in the metallized pellets continued to be reduced by graphite during melting separation, and more alloy could be obtained [29,30]. Other oxide crucibles, such as alumina, magnesium oxide, zirconia, etc., would be rapidly eroded, and the slag composition would change at a melting temperature of 1853 K.

**Figure 5:**
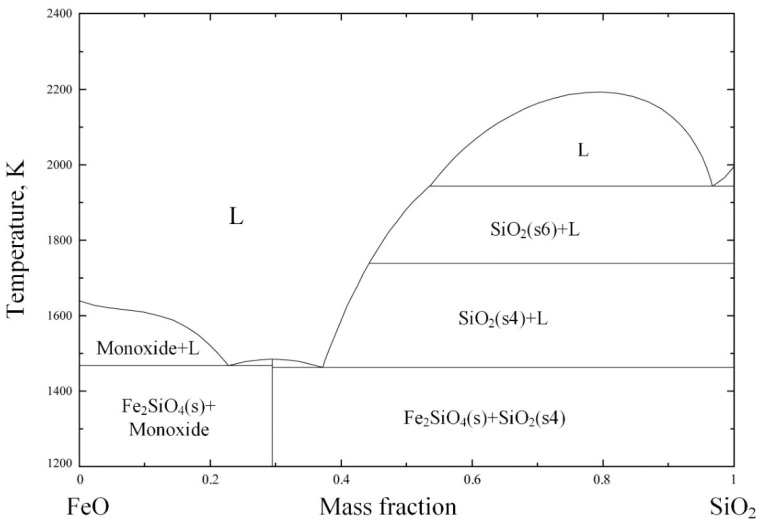
FeO–SiO_2_ phase diagram for *p* = 1 atm, drawn with FactSage 7.2.

Comparing the experimental results with different pre-reduction temperatures, it should be noted that the alloy output yield at higher temperatures (1523 K) was relatively lower, especially for the melting separation process, although the thermodynamic theoretical analysis showed that the higher the temperature, the more iron oxide would be reduced [31]. However, many previous studies have found that the low-melting-point phases in the pellet will melt during pre-reduction, which decreases the permeability of the pellets and hinders the reduction reaction. As a result, the metallization rate of the pellets is relatively lower at higher temperatures [32]. According to the FeO–SiO_2_ phase diagram in Figure 5, FeO and SiO_2_ in slag easily form low-melting-point composite oxides, with a melting temperature of only 1451 K. As mentioned above, in Experiment NO. 2, SiO_2_ was added to reduce the slag melting point to achieve a better melting separation, so the inhibition effect of a low-melting-point material on the reduction was more significant than that in Experiment NO. 4. Noting this, the FeO–SiO_2_ phase diagram was calculated and drawn by the thermodynamic software FactSage 7.2. During the phase diagram calculation, the selected database was FToxide, and the operating pressure was 101,325 Pa.

### 3.2. Iron Recovery Rate

In order to further study the transfer behaviors of iron during the processes of pre-reduction and melting/magnetic separation, the distribution of iron in the slag and alloy was assessed. The distribution ratio of iron between the slag and alloy is defined as *L_Fe_*.

*L_Fe_* = (% *Fe*)_*s*_/(% *Fe*)_*a*_ (2)

where (% *Fe*)*_s_* is the iron content in the slag after separation and (% *Fe*)*_a_* is the iron content in the alloy after separation, both measured as wt.%.

**Figure 6:**
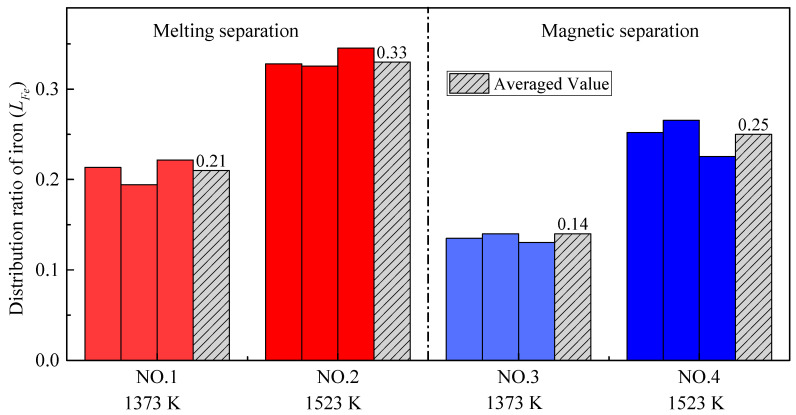
The distribution ratio of iron with different pre-reduction temperatures and different separation processes.

Figure 6 shows the distribution ratios of iron (*L_Fe_*) obtained by all four group experiments. Firstly, no matter whether melting separation or magnetic separation was used, the distribution ratio of iron at higher pre-reduction temperatures was larger. Specifically, for the melting separation process, when the pre-reduction temperature increased from 1373 K to 1523 K, the average distribution ratio of iron between the slag and alloy increased from 0.21 to 0.33. For the magnetic separation process, when the pre-reduction temperature increased from 1373 K to 1523 K, the average distribution ratio of iron increased from 0.14 to 0.25. The main reason for the above phenomenon is the formation of a low-melting-point phase. Generally, the iron oxide is mainly reduced by CO in the pellets [33,34] and the melting of the low-melting-point phase will deteriorate the permeability of the pellets, thereby hindering the reduction of iron oxides, and thus more iron remains in the slag.

Another major concern is that, when the pre-reduction temperature is the same, the distribution ratio of iron after melting separation is significantly higher than that after magnetic separation. The reduction of chromium at melting temperature can explain these results. The reduction of chromium mainly occurs in the melting separation process, while the reduction of iron mainly occurs in the pre-reduction process. Compared with the magnetic separation process, much more metallic chromium is reduced into the alloy during melting separation, leading to the decrease in iron content in the alloy, and the increase in the iron distribution ratio between the slag and alloy.

Besides the distribution ratio, the recovery rate of iron is also an important indicator. The recovery rate of iron is defined as *R_Fe_* in this paper.

(3) $$R_{Fe}=\frac{m_{a}\cdot {\left(\%\;Fe\right)}_{a}\;}{m_{0}\cdot {\left(\%\;Fe\right)}_{0}}\times \;100\%$$

where *m_a_* is the mass of obtained alloy, in g; (% *Fe*)*_a_* is the iron content of obtained alloy, in wt.%; *m*_0_ is the mass of metallized pellets before separation, in g; and (% *Fe*)_0_ is the iron content of the metallized pellets before separation, in wt.%.

**Figure 7:**
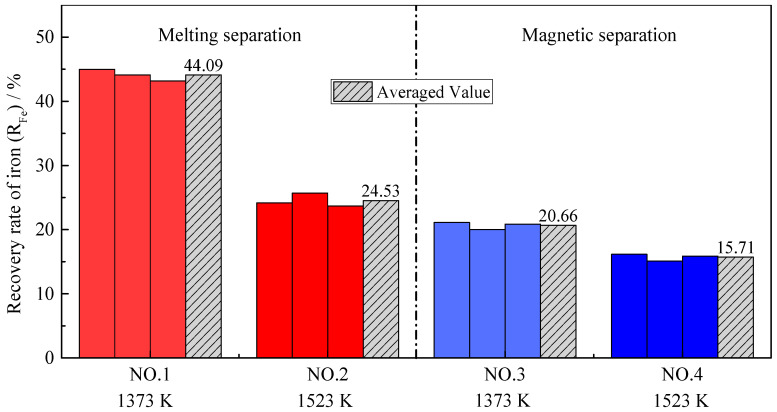
Recovery rate for iron with different pre-reduction temperatures and different separation processes.

Figure 7 shows the recovery rates of iron (*R_Fe_*) obtained by all four group experiments. The recovery rate of iron after melting separation is higher than that after magnetic separation. The results validate that the reduction of iron oxide is continuously occurring in the melting stage, leading to more iron being recovered from the slag compared to the alloy and higher recovery rate of iron. In addition, it can be seen from Figure 7 that the recovery rate of iron is relatively low at higher temperatures; the reason for this is also the melting of the low-melting-point phase, as mentioned above.

### 3.3. Chromium Recovery Rate

The distribution ratio of chromium between the slag and alloy is defined as *L_Cr_*, which is similar to the definition of the iron distribution ratio.

*L_Cr_* = (% *Cr*)_*s*_/(% *Cr*)_*a*_ (4)

where (% *Cr*)*_s_* is the chromium content in the tail slag after separation and (% *Cr*)*_a_* is the chromium content in the alloy after separation, both measured as wt.%.

Because the purpose of this research was to enrich chromium in slag, the recovery rate of chromium was defined as the ratio of the mass of chromium in the tail slag to the mass of chromium in the metallized pellets, which is different from the definition of the iron recovery rate.

(5) $$R_{Cr}=\;\frac{m_{s}\cdot {\left(\%\;Cr\right)}_{s}\;}{m_{0}\cdot {\left(\%\;Cr\right)}_{0}}\times \;100\%$$

where *R_Cr_* is the recovery rate of chromium, in %; *m_s_* is the mass of the tail slag after separation, in g; (% *Cr*)_s_ is the chromium content of the tail slag after separation, in wt.%; *m*_0_ is the mass of the metallized pellets before separation, in g; and (% *Cr*)_0_ is the chromium content of the metallized pellets before separation, in wt.%.

**Figure 8:**
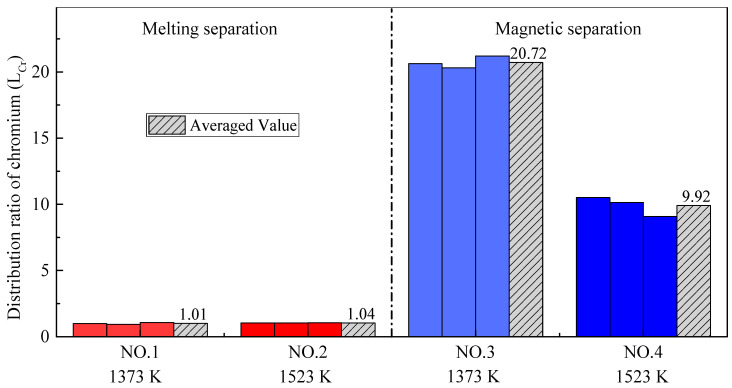
Distribution ratio of chromium with different pre-reduction temperatures and different separation processes.

**Figure 9:**
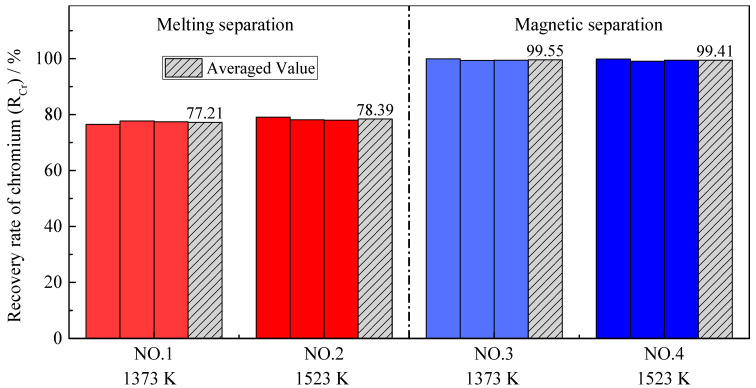
Recovery rate of chromium with different pre-reduction temperatures and different separation processes.

As shown in Figure 8 and Figure 9, the distribution ratio of chromium between the slag and alloy (*L_Cr_*) after the melting separation process was far lower than that after the magnetic separation process, while the recovery rate of chromium was also significantly lower than that after the magnetic separation process, which is different from iron. In the process of magnetic separation, only strong magnetic materials, such as metallic iron and magnetite, can be retained in the magnetic field in the magnetic separation tube, while other diamagnetic or paramagnetic materials, such as slag and residual carbon, will remain with the tail slag in the flowing water [35]. According to previously published research [36,37,38], chromium cannot be significantly reduced into metal at a temperature of 1373–1573 K, and the products obtained from magnetic separation contain almost no chromium; that is, (% *Cr*)*_a_* is very low and (% *Cr*)*_s_* is high, making *L_Cr_* in the magnetic separation process higher. As shown in Figure 9, the chromium recovery rates in Experiment NO. 3 and Experiment NO. 4 were close to 100%, indicating that chromium rarely enters the alloy, and almost all remains in the tail slag.

According to Figure 8 and Figure 9, the pre-reduction temperature has little effect on the chromium distribution ratio and the chromium recovery rate in the case of melting separation. The reason for this is that reduction of chromium occurs mainly during the melting separation stage, rather than during pre-reduction. However, in the case of magnetic separation, the effect of the pre-reduction temperature is different. Although the pre-reduction temperature has little effect on the chromium recovery rate, it has an obvious effect on the chromium distribution ratio. The phenomenon can be explained by the experimental data from Table 4. For Experiment NO. 3, the average mass of alloy *m_a_* obtained by magnetic separation was 3.70 g, and the average chromium content in the alloy (% *Cr*)*_a_* was 1.27%. For Experiment NO. 4, the average mass of alloy *m_a_* was 3.63 g, and the average chromium content in the alloy (% *Cr*)*_a_* was 2.74%. Both the mass of the alloy and the chromium content in the alloy were very small, indicating that little chromium was reduced from the slag to the alloy; most of the chromium remained in the tail slag, so the recovery rate of chromium was very high and the difference was not obvious. The distribution ratio of chromium *L_Cr_* is the ratio between the chromium content in the slag (% *Cr*)*_s_* and chromium content in the alloy (% *Cr*)*_a_*. Since (% *Cr*)*_s_* was basically unchanged, the (% *Cr*)*_a_* increased from 1.27% to 2.74%, leading the chromium distribution ratio *L_Cr_* to decrease obviously, as shown in Figure 8. This also illustrates that, as the pre-reduction temperature increases, the reduction of chromium increases slightly in the case of magnetic separation.

### 3.4. Chromium–Iron Ratio in the Tail Slag (Cr/Fe)

**Figure 10:**
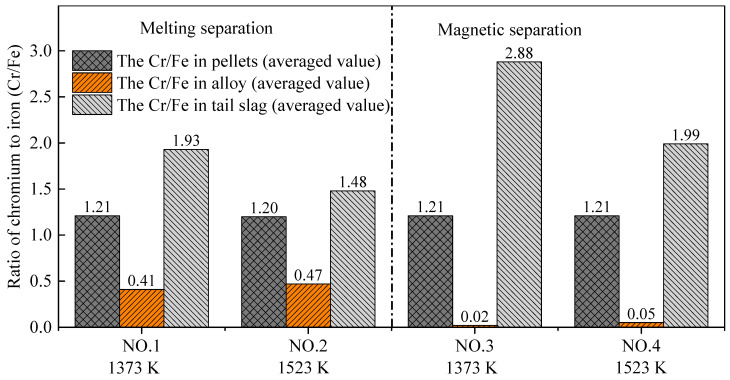
Chromium–iron ratio (Cr/Fe) in pellets, alloy, and tail slag.

As mentioned above, utilization of chromium slag requires that the chromium–iron ratio (Cr/Fe) in the tail slag is higher than 2.0. The main purpose of this research was to understand the process to achieve that ratio. The Cr/Fe ratio of the metallized pellets after pre-reduction was almost 1.2, as shown in Figure 10, which is the same as the initial raw material, according to Table 2. The Cr/Fe ratio in the alloy obtained by melting separation was obviously higher than that from magnetic separation, which is mainly due to the large amount of chromium reduced by carbon during the melting stage at 1853 K. However, the Cr/Fe ratio in the tail slag obtained by melting separation was lower than that obtained by magnetic separation. According to Table 4, Figure 8 and Figure 9, chromium mainly exists in the tail slag, and the chromium content in the tail slag changes little, although the iron content in the tail slag changes significantly.

The experimental results showed that both the melting separation and magnetic separation processes can improve the Cr/Fe ratio in tail slag. The Cr/Fe ratio in the final tail slag after the pre-reduction and separation processes decreased in the order of Experiment NO. 3, NO. 4, NO. 1, and NO. 2. Importantly, the chromium–iron ratio in the tail slag obtained by Experiment NO. 3 achieved the goal of chromium enrichment. The average Cr/Fe ratio of 2.88 was significantly higher than the critical value of 2.0.

## 4. Conclusions

In order to increase the chromium–iron ratio in the chromium slag generated by the chromite lime-free roasting process, carbothermal reduction, followed by the melting/magnetic separation process, was adopted and studied in this research. Experiments with different pre-reduction temperatures and different separation methods were carried out to investigate the specific distribution behaviors of iron and chromium between the metallic alloy and tail slag. The main conclusions are as follows:

(1) No matter whether melting separation or magnetic separation was selected, when the pre-reduction temperature increased from 1373 K to 1523 K, both the output yield of the alloy and the recovery rate of iron decreased. The analysis indicated that the melting of the low-melting-point phase decreased the permeability of the pellets and slowed down the reduction reaction. With the increase in the pre-reduction temperature, the amount of the molten phase increased and the reduction rate decreased.
(2) During the melting separation stage, because the processing temperature was higher than the reduction temperature of the iron oxides and chromium oxide, the reduction of iron further occurred and the chromium started to gradually reduce in the alloy. As a result, when the melting separation method was adopted, the output yield of the alloy, the chromium content in the alloy, and the iron recovery rate were all higher than when using magnetic separation. The melting separation process aggravated the transfer of chromium from the slag to alloy.
(3) The main purpose of this research was to increase the chromium–iron ratio in slag to more than 2.0 through a proper pre-reduction and separation process. Among the four experiments carried out, pre-reduction at 1373 K followed by magnetic separation obtained the highest chromium–iron ratio of 2.88 in the final tail slag. Simultaneously, the average recovery rate of chromium was as high as 99.55%, which met our requirements. The results indicated that a relatively low pre-reduction temperature and the magnetic separation method are beneficial for chromium enrichment in final tail slag.

## Figures and Tables

**Table 1:** Thermodynamic data for reduction reactions.

No.	Equations	ΔG^θ^ [J·mol^−1^]	Start Temperature of Reaction [K]
1	3Fe_2_O_3_ + C = 2Fe_3_O_4_ + CO	124,429 − 224.2 T	555
2	Fe_3_O_4_ + C = 3FeO + CO	207,510 − 217.62 T	953.4
3	FeO + C = Fe + CO	149,600 − 150.36 T	994.9
4	Cr_2_O_3_ + 3C = 2Cr + 3CO	771,315 − 507.11 T	1521

**Table 2:** Composition of the chromium slag used in the experiment.

T.Fe (wt.%)	T.Cr (wt.%)	Al_2_O_3_ (wt.%)	MgO (wt.%)	CaO (wt.%)	SiO_2_ (wt.%)	Cr/Fe
19.2	23.2	9.47	9.69	0.57	10.36	1.21

**Table 3:** Experimental scheme and operating parameters.

NO.	Basicity of Pellets	Pre-Reduction Temperature [K]	Pre-Reduction Time [min]	Separation Method	Separation Temperature [K]	Separation Time [min]
1	0.36	1373	45	Melting separation	1853	60
2	0.36	1523	45	Melting separation	1853	60
3	0.99	1373	45	Magnetic separation	-	-
4	0.99	1523	45	Magnetic separation	-	-

**Table 4:** Weight and composition of the metallized pellets, alloy, and tail slag.

NO.	Metallized Pellets			Alloy			Tail Slag		
	m_0_ (g)	(% Fe)_0_ (wt.%)	(% Cr)_0_ (wt.%)	m_a_ (g)	(% Fe)_a_ (wt.%)	(% Cr)_a_ (wt.%)	m_s_ (g)	(% Fe)_s_ (wt.%)	(% Cr)_s_ (wt.%)
1	91.2	18.71	22.59	13.76	55.78	22.8	70.04	11.9	22.5
	87.6	18.83	22.61	12.81	56.81	23.12	71.54	11.03	21.51
	92.1	18.67	22.8	13.65	54.33	21.89	69.1	12.04	23.51
2	91.4	18.92	22.86	8.02	52.14	24.6	64.8	17.1	25.5
	92.1	19.13	22.9	8.51	53.21	24.71	64.13	17.32	25.68
	94.5	19.79	23.58	8.5	52.13	25.32	65.31	18.01	26.61
3	60	20.11	24.21	3.78	67.42	1.27	55.4	9.1	26.2
	60	20.01	24.37	3.61	66.58	1.3	54.99	9.32	26.41
	60	20.2	24.41	3.71	68.1	1.23	55.81	8.88	26.09
4	60	20.53	25.01	3.61	55.13	2.53	56.3	13.9	26.6
	60	21.51	25.89	3.69	52.78	2.68	56.59	14.01	27.19
	60	21.72	26.11	3.59	57.61	3.01	56.9	12.99	27.36
